# Comment on Hernández-Lorca et al. Effects of Dry-Cured Ham Consumption on Cardiometabolic and Vascular Health in Adults: A Systematic Review and Meta-Analysis of Human Intervention Studies. *Foods* 2026, *15*, 1198

**DOI:** 10.3390/foods15101806

**Published:** 2026-05-20

**Authors:** Miguel López-Moreno, José Francisco López-Gil

**Affiliations:** 1 Diet, Planetary Health and Performance, Faculty of Health Sciences, Universidad Francisco de Vitoria, 28223 Madrid, Spain; 2 Institute of Health and Sport Sciences, Faculty of Health Science, Universidad Francisco de Vitoria, 28223 Madrid, Spain; 3 School of Medicine, Universidad Espíritu Santo, Samborondón 092301, Ecuador; 4 Vicerrectoría de Investigación y Postgrado, Universidad de Los Lagos, Osorno 5290000, Chile

We read with interest the systematic review and meta-analysis by Hernández-Lorca et al. [1] on the cardiometabolic effects of dry-cured ham consumption. The authors conclude that such consumption “does not appear to adversely affect conventional cardiometabolic risk markers in adults”. While this conclusion is cautiously phrased, we believe its interpretation requires further clarification regarding the underlying causal contrast.

A central issue relates to the choice and interpretation of comparators. As increasingly recognized in causal inference frameworks, dietary effects are inherently relational, reflecting substitutions rather than isolated exposures [2]. Consequently, the question addressed by an intervention is not whether a food is “neutral” or “beneficial” per se, but rather what happens when it is consumed instead of another food [3,4].

In the mentioned meta-analysis, the key randomized evidence largely compares dry-cured ham with cooked ham [5,6]. This comparator is itself a processed meat product with established limitations from a cardiometabolic perspective. Therefore, the absence of adverse effects, or even modest improvements, should be interpreted as reflecting a relative comparison between two processed meat products, rather than evidence supporting the intrinsic neutrality or healthfulness of dry-cured ham [7]. From a broader population-health perspective, it is also relevant to consider that a high processed meat (i.e., meat preserved by smoking, curing, salting, or addition of chemical preservatives) consumption remains a measurable contributor to global non-communicable disease burden. Within the Global Burden of Disease (GBD) framework, this burden is estimated relative to a theoretical minimum risk exposure level (TMREL) of 2 g/day (95% uncertainty interval [UI]: 0–4), assuming a continuous dose–response relationship without a defined threshold [8]. Accordingly, the latest GBD 2023 estimates suggest that processed meat intake was responsible for 305,756 deaths (95% UI: 138,669–507,631) and 9,845,191 (4,947,521–15,367,530) disability-adjusted life years (DALYs) worldwide [9].

Without explicitly acknowledging the comparator context, the pooled estimates may be interpreted as indicating that dry-cured ham consumption does not adversely affect cardiometabolic risk markers in general. However, most included interventions compared dry-cured ham with cooked ham rather than with minimally processed or healthier alternatives. Therefore, the meta-analysis primarily informs a relative comparison between processed meat products. Framing the findings as evidence of overall cardiometabolic neutrality extends beyond the specific comparator contrast evaluated in the included studies. As discussed in recent methodological work, statistical synthesis does not necessarily guarantee causal interpretability when pooled studies do not address an equivalent intervention contrast. In nutrition research, dietary exposures may share the same label while differing in implementation, comparator structure, or broader dietary context. Under these conditions, pooled estimates may summarize heterogeneous intervention effects rather than a single, well-defined causal contrast, limiting the interpretability of the summary estimate [2].

In addition to issues related to comparator interpretation, several methodological aspects further limit the strength of the evidence synthesis. The quantitative analysis is based on a very small number of randomized trials (*n* = 3). Although meta-analyses can be conducted with as few as two studies, statistical inference in random-effects models is often limited and of questionable value when only a few studies are available, due to low and unstable statistical power [10]. Supporting this notion, such a limited evidence base yields unstable estimates of between-study variance, making pooled effects highly sensitive to modelling assumptions rather than robust empirical signals [11,12]. This limitation is further compounded by the extremely high between-study heterogeneity observed across most outcomes (I^2^ frequently exceeding 90%), which challenges the interpretability of the summary estimates.

An additional consideration relates to crossover-specific analytical assumptions. When within-subject correlation coefficients are not reported, meta-analyses commonly rely on imputed values to estimate variance. Although this approach is methodologically acceptable and often necessary, it introduces an additional layer of uncertainty, particularly when based on a small number of studies. In such contexts, precision estimates may be influenced not only by the observed data but also by the assumptions required for synthesis [12].

Taken together, these considerations suggest that the findings may be most appropriately interpreted within the specific contrast evaluated. A more precise conclusion would be: “current evidence suggests that replacing cooked ham with dry-cured ham does not worsen short-term cardiometabolic risk markers”. Such framing more closely reflects the contrast actually evaluated and avoids extrapolation beyond the available evidence. These considerations do not invalidate the synthesis itself but may help contextualize the scope of inference supported by the available intervention evidence.

## Data Availability

The data presented in this study are available on request from the corresponding authors.
